# Pressure-Induced Structural Stabilities and Superconductivity in Rhodium Borides

**DOI:** 10.3390/ma18133125

**Published:** 2025-07-01

**Authors:** Junyi Du, Weiguo Sun, Xiaofeng Li, Xinfang Su

**Affiliations:** 1 College of Mathematical Sciences, Luoyang Normal University, Luoyang 471934, China; djy0254@126.com; 2 College of Physics and Electronic Information & Henan Key Laboratory of Electromagnetic Transformation and Detection, Luoyang Normal University, Luoyang 471934, China; xwswguo@163.com; 3 School of Science, Beijing University of Civil Engineering and Architecture, Beijing 100044, China; xfsu20002@163.com

**Keywords:** high-pressure phases, electronic structures, superconductivity, Rhodium borides

## Abstract

Transition metal borides have garnered significant research interest due to their versatile properties, including superconductivity and exceptional hardness. This study examines the stable crystal structures of Rhodium-Boron (Rh-B) compounds under high pressure using first-principles structural searching. Beyond the previously known Rh_2_B, RhB_2_, and RhB_4_ phases, three new boron-rich phases—C2/m-RhB_6_, Amm2-RhB_6_, and Cmca-RhB_8_—are identified, each characterized by three-dimensional covalent bonding networks. Their mechanical and thermodynamic stability is validated through elastic property assessments and phonon dispersion calculations. Surprisingly, these phases exhibit low bulk and shear moduli, ruling them out as candidates for hard materials. The metallic character of these borides is evident from their electronic density of states, which exhibits a sharp peak at the *E*_F_-a signature often associated with superconducting systems. Indeed, our calculations predict *T*_c_ values of 8.93 K and 9.36 K for Amm2-RhB_6_ and Cmca-RhB_8_, respectively, at 100 GPa.

## 1. Introduction

Transition metal borides have attracted a lot of attentions for their facile synthesis at ambient pressure, superior mechanical properties, and good electrical conductivity. Transition metals have rich valence electrons and easily combine with Boron atoms to promote realization of new materials with remarkable properties under extreme conditions. The existing B-B directionally strong covalent bonding patterns and high valence electron density (VED) strengthen the hardness and thermal stability of transition metal borides. Especially, transition metal borides with rich boron content, such as ReB_2_ [1], OsB_2_ [2], RuB_2_ [3], YB_4_ [4], MnB_4_ [5,6], WB_4_ [7], CrB_4_ [8,9], and others, can be experimentally synthesized at low pressure. This reduces synthesis costs and facilitates their application in various fields. In these borides, MgB_2_ [10,11] has been discovered to have the highest superconducting critical temperature (*T*_c_) (~39 K) at ambient pressure. Low compressibility is found in OsB_2_, MnB_4_, and WB_4_ [12,13,14] due to their exceptionally high shear modulus. ReB_2_ and WB_4_ have high hardnesses up to 48 GPa and 46.2 GPa, respectively [15,16]. For these reasons, many scientists have dedicated themselves to the research of transition metal borides, aiming to discover new borides with outstanding properties. Consequently, exploring novel superhard transition metal boride materials remains essential.

The Rhodium-Boron (Rh-B) system is an intriguing area of study within the field of high-pressure material science. Rhodium (Rh) is a rare, highly corrosion-resistant transition metal, while boron (B) is a metalloid with exceptional hardness and a high melting point. The combination of Rh and B can potentially lead to materials with unique physical and chemical properties suitable for advanced technological applications. Recent developments in vacuum-based electron beam techniques have yielded phase-pure hexagonal RhB1.1, providing new opportunities for studying its fundamental properties. It can be characterized by X-ray diffraction and micro-indentation techniques. Hardness values exhibit a pronounced load dependence, increasing from 7 GPa (0.49 N) to 22.6 GPa (9.81 N) in Vickers tests [17]. Additionally, a 1.0 μm thick RhB1.1 film with a high hardness of 44 GPa [18] has been found, which means it can be considered as a potential superhard material. RhB initially crystallizes in the anti-NiAs structure at atmospheric pressure, transforming into the FeB-type phase above 22 GPa [19]. The stable phases of RhB_2_ and Rh_2_B, as low compressible materials, are also established by theoretical calculation under pressure [20]. Boron-rich RhB_4_ has been investigated with the orthorhombic *Pnnm* phase. However, there is no phase transition observed in RhB_4_ up to 100 GPa [21]. In addition, the mechanical and electronic properties of Rh_5_B_4_ under pressure have also been studied [22].

As we know, the content of boron in transition metal borides is very important to their crystal structures and physical properties under pressure. The pressure could change the bonding characterization of atoms and stimulate the formation of unusual stoichiometric compounds. Still, there is scant knowledge on Rhodium borides with richer B content, although some borides (such as ReB_2_ [1], WB_4_ [2], and so on) have been well studied. The rich B atoms of stable phases easily form a complex three-dimensional network, which benefits their unique physical properties. So far, the high-pressure phase stabilities of B-rich Rh-B compounds have not been discussed. Therefore, it is very important to investigate the stability of B-rich Rh-B system under high pressure.

To find stable phases of Rh-B compounds under pressure, the phase stabilities and electronic and mechanical properties of Rh*_x_*B*_y_* system (*x* = 2, *y* = 1; *x* = 1, *y* = 1–8, 10, 12) under pressures of 0–200 GPa were studied. The exploration of crystal structures was based on the particle swarm optimization algorithm combined with first-principles calculations. In Rh-B compounds, the structures of RhB (*P*6_3_/*mmc* phase) and Rh_2_B (*Pnna* phase) synthesized in experiments [23,24] were well reproduced by our calculations. However, in our calculations, new stable stoichiometries of RhB_6_ and RhB_8_ were found under pressure. The detailed structure features, electronic properties, and mechanical properties of stable phases in Rh-B systems were investigated under pressure.

## 2. Computational Methods

In this work, structure searches for Rh-B system were employed at 0 GPa, 100 GPa, and 200 GPa by the CALYPSO code based on the particle swarm optimization methodology [25,26]. The accuracy and efficiency of CALYPSO (version 7.0) have been validated through successful structure predictions for a wide range of compounds [27,28,29]. The computational investigations were carried out employing the VASP software package (version 5.4.4.18Apr17) [30], where structural optimizations and energy calculations were performed within the GGA-PBE exchange-correlation function [31]. A plane-wave cutoff energy of 650 eV was employed to ensure convergence of the electronic structure. The 4*d*^8^5*s*^1^ and 2*s*^2^2*p*^1^ were treated as the valence electrons for Rh and B, respectively. A convergence threshold of energy of 10^−5^ eV/atom and force of 10^−3^ eV/Å were set in the optimization process. Monkhorst-Pack *k*-point meshes with grid spacings of 0.04 Å^−1^ and 0.02 Å^−1^ were employed for structural optimization and self-consistent energy calculations, respectively. The dynamical stability was evaluated through phonon calculations employing the supercell-based finite displacement method as implemented in the Phonopy package (version 2.41.0) [32]. The elastic constants were determined using the strain-energy method, which involves applying small deformations to the equilibrium lattice and fitting the resulting energy-strain relationship. Based on the Voigt-Reuss-Hill approximation, we derived the key mechanical parameters: bulk modulus, shear modulus, Young’s modulus, and Poisson’s ratio [33]. The Vickers hardness (*H*_v_) was calculated by employing Chen et al.’s empirical model [34] based on the computed elastic moduli.

The phonon-mediated superconducting properties were computed within the framework of density-functional perturbation theory (DFPT) using a plane-wave pseudopotential approach, as implemented in the Quantum ESPRESSO package (version 7.0) [35]. In the calculations, a kinetic cutoff energy of 90 Ry was used by a series of tests, which established the convergency of energy. A Q-mesh of 2 × 4 × 2 and *k*-mesh of 8 × 16 × 8 for *P*2_1_/*m*-Rh_2_B, q-mesh of 4 × 4 × 4 and *k*-mesh of 16 × 16 × 16 for *C*2/*m*-Rh_2_B and *P*6_3_/*mmc*-RhB, q-mesh of 4 × 4 × 2 and *k*-mesh of 16 × 16 × 8 for *C*2/*m* and *Amm*2-RhB_6_, and *k*-mesh of 16 × 16 × 16 for *C*2/*m*-Rh_2_B and *P*6_3_/*mmc*-RhB were used in the first Brillouin zone (BZ) for the electron–phonon matrix calculations. The superconducting temperature *T*_c_ of stable phases of Rh-B compounds was calculated via the Allen–Dynes-modified McMillan equation [36].

## 3. Results and Discussion

To find stable structures of Rh-B compounds under pressure, the stoichiometries of the Rh*_x_*B*_y_* system (*x* = 2, *y* = 1; *x* = 1, *y* = 1–8, 10, 12) with cells of 1–4 formula units (f.u.) in size are fully explored up to 200 GPa by CALYPSO code [25,26]. In order to study the thermodynamical stability of the Rh-B system, the formation enthalpies were calculated as

$$∆H=[H\left({Rh}_{x}B_{y}\right)-xH\left(Rh\right)-yH\left(B\right)]$$

**Figure 1:**
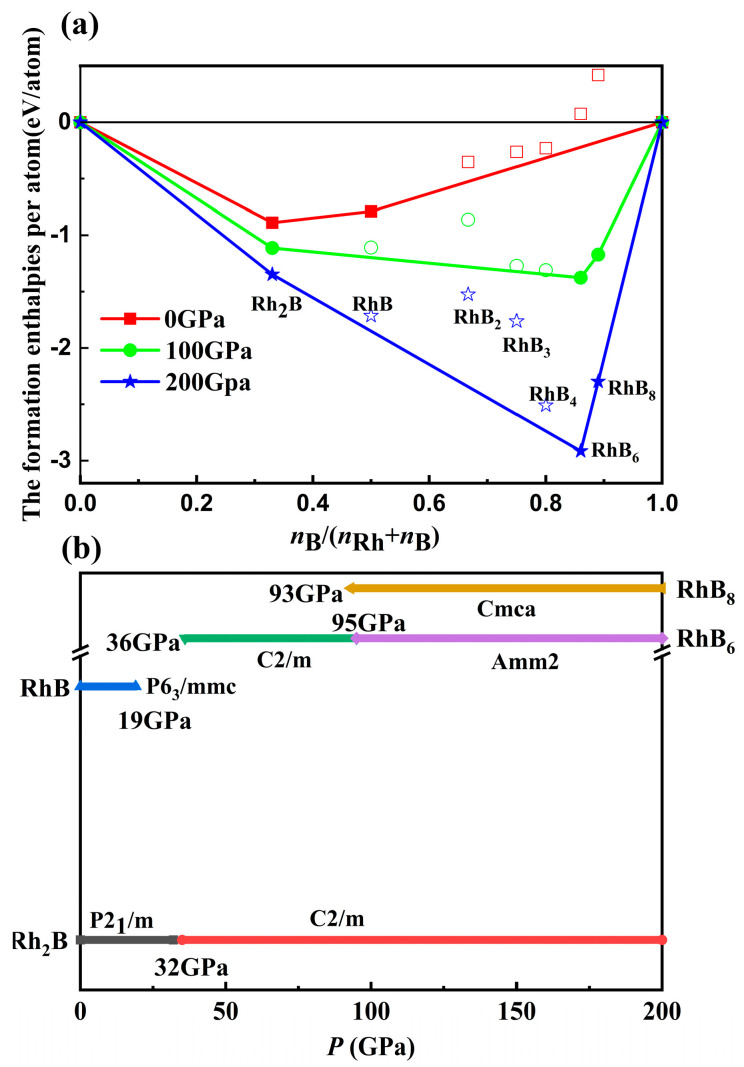
(**a**) The relative enthalpies of formations as a function of atomic B content for the selected Rh*_x_*B*_y_* system phases at different pressures. (**b**) Phase diagram region of Rh-B under pressures.

Here, *H* (Rh*_x_*B*_y_*), *H* (Rh), and *H* (B) are the enthalpies of Rh*_x_*B*_y_*, Rh, and B, respectively. The cubic phase of Rh [37] and the hexagonal α-B [38] are established as the referential phases. Based on the definition of formation enthalpies of Rh-B compounds, the phase stabilities of various stoichiometries in the Rh-B system are displayed in Figure 1a, in which the convex hull is constructed relative to solid Rh and B. The dots on the solid line indicate thermodynamic stability, while the hollow symbols above the convex hull represent metastable structures in the Rh-B system. The most stable structure corresponds to the lowest formation enthalpy against decomposing into Rh and B at the given pressure. As showed Figure 1a, the formation enthalpies for Rh-B compounds are negative except RhB_6_ and RhB_8_ at 0 GPa. Rh_2_B and RhB were located on the solid line of the convex hull, which indicated their thermodynamic stability. With increasing pressure up to 100 GPa, RhB unexpectedly became unstable and disappeared from the solid line. Surprisingly, thermodynamic stability was found for two new compounds, RhB_6_ and RhB_8_, which indicated that RhB_6_ and RhB_8_ could be experimentally synthesized by metal Rh and B. As pressure continued to increase up to 200 GPa, the compounds remained stable. In order to establish the phase transition, Figure 1b exhibits the pressure ranges of the stable compounds.

**Figure 2:**
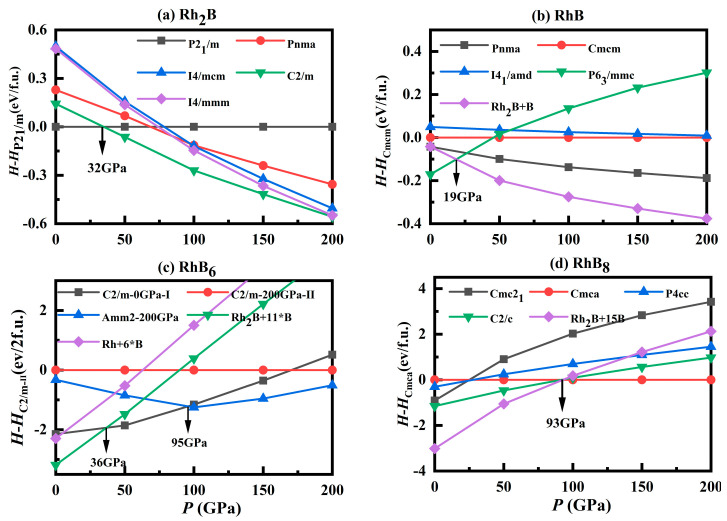
Calculated enthalpies per atom of various structures relative to corresponding predicted phases for stable Rh-B phases in the pressure range of 0–200 GPa. The star means the time of B atoms.

**Figure 3:**
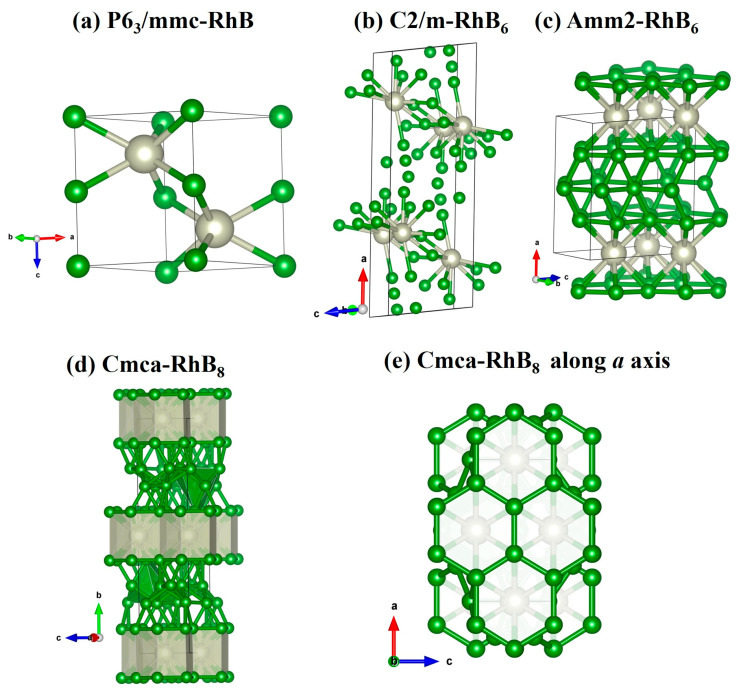
The crystal structures and atomic arrangements of predicted stable Rh-B compounds. The green balls represent B atoms, and the white balls represent Rh atoms.

For Rh_2_B, pressure-dependent phase transitions from the *P*2_1_/*m* to *C*2/*m* phase were observed at 29 GPa (Figure 2a), which is consistent with Ref. [19]. RhB crystalizes in the hexagonal *P*6_3_/*mmc* phase (Figure 3a) and keeps stable below 19 GPa (Figure 2b). The predicted new monoclinic *C*2/*m*-RhB_6_ could be stable above 36 GPa (Figure 2c), which could be experimentally synthesized by Rh_2_B and element B. Under high pressure, the monoclinic phase *C*2/*m* transforms into the orthorhombic *Amm*2 phase at 95 GPa. The *C*2/*m* phase structures feature four molecules per unit cell, including the RhB_10_ structural unit. From Figure 3b, the B atoms in the RhB_10_ structural unit of the *C*2/*m* phase are not evenly distributed around the Rh atom. Under high pressure, the number of boron atoms surrounding the Rh atom increases from 10 to 12. The orthorhombic *Amm*2 phase consists of RhB_12_ polyhedra, where each Rh atom is connected to 12 neighboring B atoms (Figure 3c). Notably, it exhibits an interesting RhB_12_-B-RhB_12_ sandwich stacking order. Along the a-axis, the crystal structure exhibits alternating B hexagonal planes interconnected through RhB_12_ hexagonal columns, forming a three-dimensional framework. So far, the metal hexa-borides have exhibited a wide range of unusual physical properties, which mainly contributed to their crystal phases [39,40,41]. The new predicted stable crystal phases of RhB_6_ are the same at those of ZrB_6_ under pressure, for which their B atom arrangement is also similar. For RhB_8_, it stabilizes as the orthorhombic *Cmca* phase above 93 GPa, which has no phase transition under higher pressure (Figure 2d). Figure 3e shows the *Cmca* phase RhB_12_ polyhedral and B parallel hexagonal planes similar to *Amm*2-RhB_6_. However, there are two-layer zigzag B_4_ chains between two RhB_12_ hexagonal columns. The structural information of the new predicted phases is described in Table 1.

**Figure 4:**
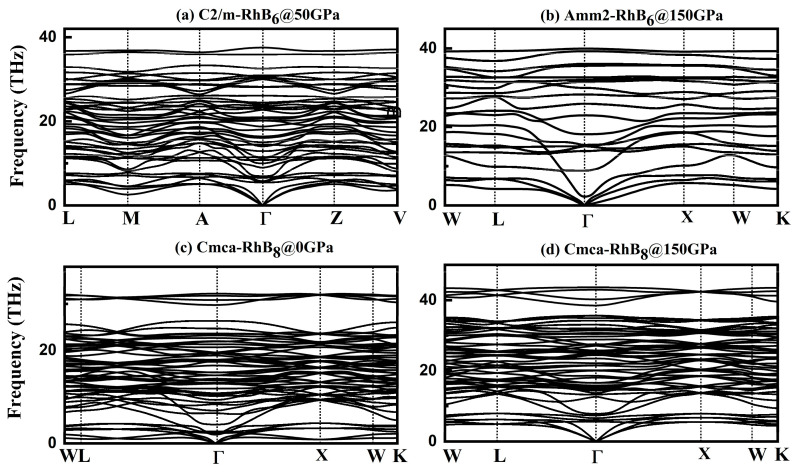
The phonon spectrum of predicted stable phases *C*2/*m*-, *Amm*2-RhB_6_, and *Cmca*-RhB_8_ under pressure.

All positive phonon frequencies indicate the stability of the crystals. Phonon dispersion calculations performed via the finite displacement method confirm the dynamical stabilities of predicted RhB_6_ and RhB_8_. No imaginary modes were found in the phonon frequencies of RhB_6_ and RhB_8_ within their stability pressure ranges (Figure 4). Notably, orthorhombic RhB_8_ exhibited no imaginary phonon modes in the BZ at ambient pressure, suggesting its potential recoverability under ambient conditions.

Elastic parameters provide fundamental insights into key mechanical properties, including phonon dispersion relations, bonding characteristics, and mechanical stability criteria. Meanwhile, they provide valuable information for estimating material hardness. The elastic parameters have been listed in Table 2. Moreover, as seen from Table 2, all four phases of RhB compounds are stable, except for *Amm*2-RhB_6_ at ambient pressure, according to mechanical stability criteria [42].

From Table 2, the largest values of *C*_11_ in *P*2_1_/*m*-, *C*2/*m*-Rh_2_B, *P*6_3_/*mmc*-RhB, *C*2/*m*-, *Amm*2-RhB_6_, and *Cmca*-RhB_8_ reveal their higher linear incompressibility along the *a*-axis than that along *b*- and *c*-axes. *Amm*2-RhB_6_ and *Cmca*-RhB_8_ have the largest *C*_33_ values, indicating their low compressibility along the *c*-axis. Meanwhile, the estimated bulk modulus *B*, shear modulus *G*, and Young’s modulus *E* are also consistent with other calculated data [17,19,42,43]. In comparison to ReB_2_ and ZrB_6_, the stable Rh-B compounds have lower values of *C*_11_, *C*_22_, *C*_33_, and shear modulus, which maybe play important roles in their compressibility. Hardness is another important parameter representing the mechanical properties of materials. The hardness of stable Rh-B compounds under ambient conditions has been successfully calculated and is listed in Table 2 using an empirical model recently proposed by Chen et al. [34]. Notably, the stable Rh-B phases display unexpectedly low hardness values compared to isostructural transition metal borides. The calculated low hardness of these stable phases aligns with their relatively small Young’s modulus and large Poisson’s ratio. A low Young’s modulus indicates a weak ability to resist tension and pressure within the range of elastic deformation, while the large Poisson’s ratio suggests weak directional covalent bonding, which reduces the material’s hardness.

**Figure 5:**
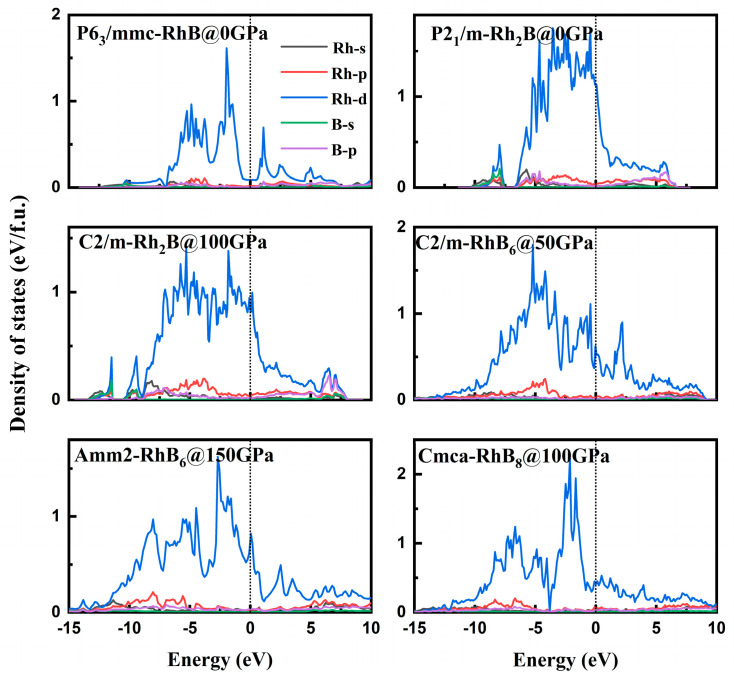
The partial densities of states for stable Rh-B compounds under pressure.

**Figure 6:**
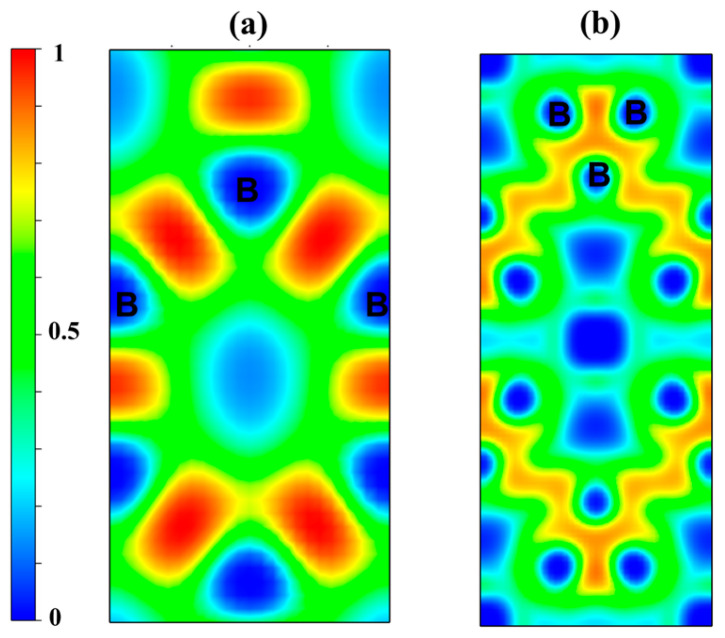
Contours of the electronic localization function (ELF). Electron localization function isosurface maps for (**a**) (100) plane of *Amm*2-RhB_6_ and (**b**) (001) plane of *Cmca*-RhB6 at 150 GPa.

In order to investigate the electronic properties of stable Rh-B compounds, their partial densities of states (PDOSs) are investigated under pressure. As shown in Figure 5, all thermodynamically stable Rh-B phases exhibit finite densities of states at the Fermi level (*N*(*E*_F_)), confirming their metallic character. The density of states of Rh-*d* and B-2*p* occupied most of total density of states at the Fermi level. Moreover, the similar shapes of the density of states of these stable compounds were observed in the whole energy range, exhibiting hybridizations between Rh-3*d* and B-2*p* orbitals. Notably, stable Rh-B compounds lacked the characteristic deep pseudo gap valleys near the Fermi level, which were evidenced by their finite density of states across the entire energy range. Therefore, the relatively weak covalent bonding formed by Rh-3*d* and B-2*p* to other borides decreases their hardness. The decrease of the total DOS at the Fermi level for *Amm*2-RhB_6_ and Cmca-RhB_8_ is accompanied by the appearance of the pseudogap around the Fermi level, which is helpful to increase the stability of orthorhombic RhB_8_. To further investigate the chemical bonding in RhB_6_ and RhB_8_, electron localization functions (ELFs) were calculated to help visualize B-B bonding. A high ELF (0.5–1) indicates that the electrons are highly localized and strong covalent bonds exist, while a low ELF (0–0.5) demonstrates that the electrons are highly delocalized and ionic bonds exist. Figure 6 displayed ELFs for the (100) plane of *Amm*2-RhB_6_ and (001) plane of *Cmca*-RhB_8_ at 150 GPa. Strong covalent bonding of B-B in *Amm*2-RhB_6_ and appreciably weaker B-B bonding in *Cmca*-RhB_8_ was observed. The strong covalent bonding helps to increase the stability of RhB_6_ and RhB_8_.

Based on their metallicity and electronic structural characteristics of stable Rh-B compounds under pressure, electron-coupling calculations were applied to investigate the superconductivity of Rh_2_B, RhB, RhB_6_, and RhB_8_ at different pressures. It was applied to different compounds [45,46] to indicate the reasonability of the calculations. Table 3 exhibited the relevant superconducting parameters the EPC parameters λ, logarithmic average frequency ω_log_, the values of density of states at the Fermi level, and superconducting temperature *T*_c_ at different pressures. The results indicated that Rh_2_B, RhB, RhB_6_, and RhB_8_ have quite low superconducting temperatures close to zero under high pressure due to the low logarithmic average frequency and electron–phonon coupling parameter. *C*2/*m*- and *Amm*2-RhB_6_ at 100 GPa has 8.93 K and 9.36 K with EPC parameters of λ 0.505 and 0.566, respectively, in which λ plays an important role in the superconducting temperature in transition borides. Though RhB_6_ has a similar layered structures to that of MgB_2_, it has a low *T*_c_ in comparison to MgB_2_ [47].

**Figure 7:**
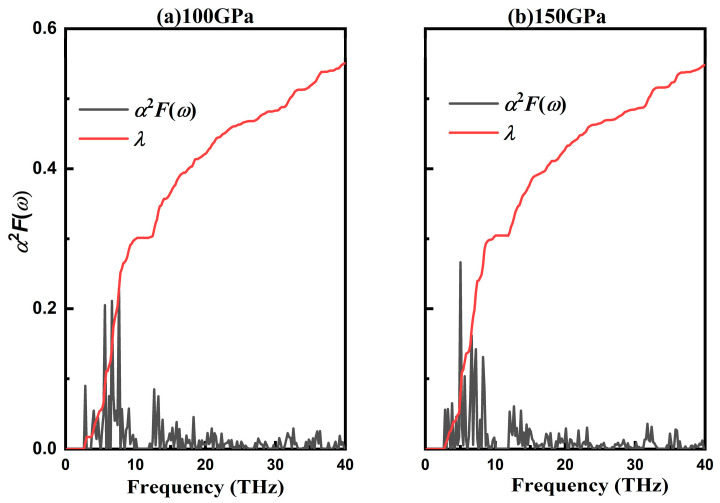
Eliashberg spectra function and electron–phonon coupling parameter at 100 GPa and 150 GPa of *Amm*2-RhB_6_.

In order to the identify the effect of pressure on the superconducting temperature, the *T*_c_ of RhB_8_ under pressure was calculated. The density of states of RhB_8_ at the Fermi level decreased from 0.685 at 100 GPa to 0.608 at 200 GPa, and the logarithmic average frequency ω_log_ was 616.2 K at 100 GPa and 705.8 K at 200 GPa. With pressure increasing from 100 GPa to 20 GPa, the EPC parameter in RhB_8_ decreased from 0.528 to 0.450, which led to the superconducting temperature changing from 9.36 K to 5.45 K. In order to investigate the superconducting mechanism of the Rh-B system, Figure 7 presents the Eliashberg EPC spectral function α^2^F(ω) and its integral of Amm2-RhB_6_ at 100 and 150 GPa. It is seen that the phonon modes are separated into two parts: the acoustic phonon modes (low frequencies < 10 THz) dominated by vibrations of Rh atoms and the optical phonon modes (high frequencies) from the vibrations of B atoms. When the pressure increases from 100 GPa to 150 GPa, the electron–phonon coupling parameter decreases from 0.56 to 0.55, indicating the low influence of pressure on the superconducting transition temperature. The underlying mechanisms stimulate more comprehensive exploration for the superconducting properties of transition metal borides.

## 4. Conclusions

In conclusion, first-principles structural searching was extensively explored to obtain the stable crystal structures of Rh-B compounds under high pressure. The stable pressure ranges of the stable Rh-B phases were established. Based on the previously predicted Rh_2_B, RhB_2_, and RhB_4_, three new phases of the boride-rich compounds RhB_6_ and RhB_8_ (*C*2/*m*-RhB_6_, *Amm*2-RhB_6_, and *Cmca*-RhB_6_) were established under pressure for the first time. Three-dimensional covalent bonding networks were found in the boride-rich RhB_6_ and RhB_8_. Based on their elastic properties and the calculation of phonon dispersion, the *C*2/*m*-, *Amm*2-RhB_6_, and *Cmca*-RhB_8_ were found to have mechanical and thermodynamic stability. Surprisingly, the three stable phases of RhB_6_ and RhB_8_ had a low bulk and shear modulus, and the hardness was not regarded as indicating potential hard materials. Meanwhile, the electronic structures and densities of states of the stable phases of Rh-B under pressure were also investigated. From the shapes of the densities of states, no deep pseudo-gap valleys near the Fermi level were seen, similar to other high-hardness transition metal borides. Electronic structure calculations revealed substantial densities of states at *E*_F_ for the stable Rh-B phases, demonstrating their metallic character. Surprisingly, the phonon dispersion and electron–phonon coupling calculations suggest that *Amm*2-RhB_6_ and *Cmca*-RhB_8_ at 100 GPa have superconducting temperatures of 8.93 K and 9.36 K, respectively. For other stable Rh-B compounds, their superconducting temperatures are very low, close to zero. Moreover, the superconducting temperatures decrease with pressure for RhB_6_ and RhB_8_. The existence of superconducting transition metal borides will provide diversity to the family of superconductors. The establishment of phase regions of stable Rh-B compounds provides a synthesized route of transition for metal borides under certain extreme conditions. The findings will benefit from future theoretical and experimental research in the field.

## Figures and Tables

**Table 1:** The calculated optimized equilibrium lattice parameters *a*, *b*, and *c* and the atom position of stable phases of the Rh-B system at different pressures.

	*P* (GPa)	Lattice Constants (Å)	*x*	*y*	*z*	Site
*P*2_1_/*m*-Rh_2_B	0	*a* = 5.587	0.926	0.250	0.231	Rh (2e)
		*b* = 2.813	0.563	0.750	0.770	Rh (2e)
		*c* = 4.684	0.776	0.250	0.604	B (2e)
*C*2/*m*-Rh_2_B	50	*a* = 8.429	0.421	0	0.095	Rh (4i)
		*b* = 2.722	0.287	0	0.360	Rh (4i)
		*c* = 7.073	0.123	0	0.652	B (4i)
*P*6_3_/*mmc*-RhB	0	*a* = *b* = 3.378	0.667	0.333	0.75	Rh (2a)
		*c* = 4.140	0	0	0	B (2c)
*C*2/*m*-RhB_6_	0	*a* = 14.01	0.191	0.5	0.152	Rh (4i)
		*b* = 2.777	0.288	0.5	0.514	B (4i)
		*c* = 4.821	0.919	0.5	0.177	B (4i)
			0.962	0.5	0.836	B (4i)
			0.359	0.5	0.262	B (4i)
			0.098	0	0.348	B (4i)
			0.982	0	0.326	B (4i)
*Amm*2-RhB_6_	150	*a* = 5.688	0	0.5	0.408	Rh (2a)
		*b* = 2.567	0.261	0.5	0.071	B (4c)
		*c* = 5.245	0.252	0	0.258	B (4c)
			0.5	0	0.103	B (2b)
			0.5	0	0.408	B (2b)
*Cmca*-RhB_8_	100	*a* = 5.288	0.5	0	0.5	Rh (4a)
		*b* = 13.271	0.5	0	0.007	B (16g)
		*c* = 2.908	0.75	0.711	0.25	B (8e)
			0.5	0.218	0.948	B (8f)

**Table 2:** The elastic constants *C*_ij_ (GPa), bulk modulus *B* (GPa), shear modulus *G* (GPa), Young’s modulus *E* (GPa), and hardness *H*_V_ (GPa); Poisson’s ratio ν of stable Rh-B compounds at zero pressure; and other theoretical data.

Structures		*C*_11_	*C*_22_	*C*_33_	*C*_44_	*C*_55_	*C*_66_	*C*_12_	*C*_13_	*C*_23_	*B*	*G*	*E*	*ν*	*H*_V_
*P*2_1_/*m*-Rh_2_B	this work	483	348	363	81	75	57	158	154	189	243	84	227	0.344	4.7
*P*2_1_/*m*-Rh_2_B	Ref. [17]	339	350	527	56	57	73	187	143	132	238	87	232	0.337	
*C*2/*m*-Rh_2_B	this work	506	328	369	64	53	50	148	138	198	239	74	202	0.360	3.3
*P*6_3_/*mmc*-RhB	this work	439		303	155			204	247		283	106	282	0.334	7.6
	Ref. [19]	438		342	172			223	256		296	102			
*C*2/*m*-RhB_6_	this work	467	448	360	123	34	145	128	181	94	288	143	354	0.242	13.6
*Amm*2-RhB_6_	this work	487	330	501	151	65	130								
*Cmca*-RhB_8_	this work	392	472	515	15	82	116	190	133	159	258	56	156	0.399	2.3
*P*6_3_/*mmc*-ReB_2_	Ref. [43]	643		1035	263			159	129		344	364	642	0.21	
*Amm*2-ZrB_6_	Ref. [44]	683	698	623	215	295	125	65	94	101	280	230	543	0.178	40.1
*Cmcm*-ZrB_6_	Ref. [44]	660	669	601	220	283	139	66	86	94	269	231	540	0.165	24.6

**Table 3:** Predicted electron–phonon coupling and superconducting properties of stable Rh-B compounds at pressure. *T*_c_ is estimated by Allen–Dynes-modified McMillan equation.

	Pressure (GPa)	*N*_f_ (states/ev/f.u.)	*ω*_log_ (K)	*λ*	*T*_c_ (K)
*P*2_1_/*m*-Rh_2_B	0	1.182	221.8	0.411	1.08
	50	0.971	291.2	0.335	0.37
*C*2/*m*-Rh_2_B	50	1.005	246.8	0.406	1.22
*P*6_3_/*mmc*-RhB	0	0.189	281.8	0.251	0.03
*C*2/*m*-RhB_6_	50	0.619	506.7	0.408	2.39
*Amm*2-RhB_6_	100	0.662	468.7	0.566	8.93
	150	0.608	494.2	0.505	6.32
*Cmca*-RhB_8_	100	0.685	616.2	0.528	9.36
	150	0.639	698.5	0.473	6.92
	200	0.608	705.8	0.450	5.45

## Data Availability

The original contributions presented in this study are included in the article. Further inquiries can be directed to the corresponding author.
